# Quantum probability in decision making from quantum information representation of neuronal states

**DOI:** 10.1038/s41598-018-34531-3

**Published:** 2018-11-01

**Authors:** Andrei Khrennikov, Irina Basieva, Emmanuel M. Pothos, Ichiro Yamato

**Affiliations:** 1 International Center for Mathematical Modeling in Physics and Cognitive Sciences Linnaeus University, Växjö, S-35195 Sweden; 2 National Research University of Information Technologies, Mechanics and Optics (ITMO), St. Petersburg, Russia; 3 Department of Psychology, City, University of London, London, UK; 4 Department of Biological Science and Technology, Tokyo University of Science, 2641 Yamazaki, Noda-shi, Chiba, 278-8510 Japan; 5 Department of Chemistry, Graduate School of Science, Chiba University, 1-33 Yayoi-cho, Inage, Chiba, 263-8522 Japan

## Abstract

The recent wave of interest to modeling the process of decision making with the aid of the quantum formalism gives rise to the following question: ‘How can neurons generate quantum-like statistical data?’ (There is a plenty of such data in cognitive psychology and social science). Our model is based on quantum-like representation of uncertainty in generation of action potentials. This uncertainty is a consequence of complexity of electrochemical processes in the brain; in particular, uncertainty of triggering an action potential by the membrane potential. Quantum information state spaces can be considered as extensions of classical information spaces corresponding to neural codes; e.g., 0/1, quiescent/firing neural code. The key point is that processing of information by the brain involves superpositions of such states. Another key point is that a neuronal group performing some psychological function *F* is an open quantum system. It interacts with the surrounding electrochemical environment. The process of decision making is described as decoherence in the basis of eigenstates of *F*. A decision state is a steady state. This is a linear representation of complex nonlinear dynamics of electrochemical states. Linearity guarantees *exponentially fast convergence to the decision state*.

## Introduction

The recent revolution in quantum information has a strong foundational impact, in particular, development of the quantum information interpretation (Zeilinger and Brukner^1^, Plotnitsky^2^, D’ Ariano^3^, Fuchs and Schack^4^). According to this interpretation, the quantum formalism is about information and probability which can be gained through observations (cf. Bohr^5^). In principle, the quantum formalism endowed with the information interpretation can be applied to other areas of research^6^, e.g., modeling the process of decision making by humans and, more generally, human cognition. We remark that the same conclusion can be derived on the basis of consistent consideration of the principles of quantum logic: violation of the laws of Boolean logic needs not be rigidly coupled to observation on quantum physical systems (see especially Svozil^7^ and Ozawa^8,9^).

We now turn to theory of decision making and recall that Tversky and Kahenman^10^ and other researchers in psychology and economics (starting with the seminal paradoxes of Allais^11^ and Ellsberg^12^) demonstrated cases where *classical probability* (CP) prescription and actual human thinking persistently diverge, at least relative to baseline classical intuitions. There is a plenty of probabilistic data that do not match the laws of CP. These data was typically related to probability fallacies and irrational behavior.

*Do people follow the CP-rules*? *Are there any other laws that can be applied to formalize human judgments*?

After demonstrating first evidence on deviation from the postulates of CP-based decision models, Tversky, Kahenman started to advertise the heuristic approach as an alternative to probabilistic modeling of decision making.

However, as was shown during the last ten years, some of the main problems of the CP-based decision making, expected utility theory and its later modifications such as subjective expected utility and prospect theories, can be resolved on the basis of *quantum probability* (QP) calculus. The QP-approach to modeling of decision making is a purely operational approach describing probability distributions of observations’ outputs. The description is formal and it is based on the calculus of Hermitian operators in complex Hilbert space.

Researchers use the quantum formalism to describe aforementioned nonclassical data, to resolve paradoxes and to model various psychological effects such as conjunction, disjunction, and order effects, see, e.g., monographs^6,13–15^ and some representative papers^16–21^. The main tool is the machinery of quantum interference for incompatible observables^6,14^; see also^22,23^ for tests of contextuality in decision making based on the Bell-type^24,25^ inequalities. In many cases, models based on quantum theory can be seen as providing a formalization of relevant heuristic principles (e.g., the quantum model for the *conjunction fallacy*^18^, can be thought of as a formalization of Tversky and Kahneman’s *representativeness heuristic*^26^).

As is often the case with cognitive models^27^, in this approach the brain is considered as *a black box* that processes information in accordance with the laws of quantum information theory and generates QP-data. To distinguish this operational approach from the approaches based on quantum physical processes in the brain (e.g.^28,29^), we call it the *‘quantum-like’*. The quantum-like community is multi-disciplinary: around two hundred experts in cognitive, social, and political sciences, psychology, quantum physics, economics, finances, genetics, and molecular biology. For the expression ‘quantum-like’, Google scholar gives 5260 links. However, the absence of connection with neurophysiological processes makes the grounds of the quantum-like modeling rather shaky. There is a deep gap between neurophysiology and the quantum-like approach or cognitive informatics in general. The aim of this paper is to make this gap less deep, cf.^30,31^.

From the philosophical viewpoint, we handle the problem of reduction of mental processes to electrochemical processes at the neuron level. This reduction is not straightforward and it is based on the methodology of the *ontic-epistemic approach*, see Atmanspacher^32^, see also^33^.

## Results

### Modeling information processing by neurons with theory of open quantum systems

The main idea behind our model connecting functioning of neuronal structures to quantum-like statistics of decision making by humans is that neuron’s state space must have the Hilbert space structure, i.e., a single neuron has to ‘work’ with probabilistic *superpositions* of states caring cognitive information. At the same time a neuron is treated as an open system, which state of superposition is affected by information flows from the electrochemical environment composed of signals (electrical currents, electromagnetic field, neurotransmitters) generated by other neurons.

Authors developing the genuine quantum physical models of the brain functioning pointed to impossibility of considering neurons in a state of superposition^28,29^. As a consequence, neurons were not considered as the basic units of information processing. This viewpoint on neurons’ role in information processing diminished the interest of neurophysiologists to such models. In our model, we present a natural electrochemical basis for superposition states of a neuron or a group of neurons (in the latter case, states are generally non-separable - entangled).

The classical information approach to modeling of the brain functioning uses discrete states of a neuron corresponding to various neural codes^34^; e.g., 0/1, *quiescent/firing neural code*. We stress the role of states of uncertainty, e.g., *neither quiescent nor firing*, in information processing. Such states can be represented as superpositions. Our aim is to lift processing of such states to the quantum-like statistics of outputs of a psychological function *F*.

**Figure 1:**
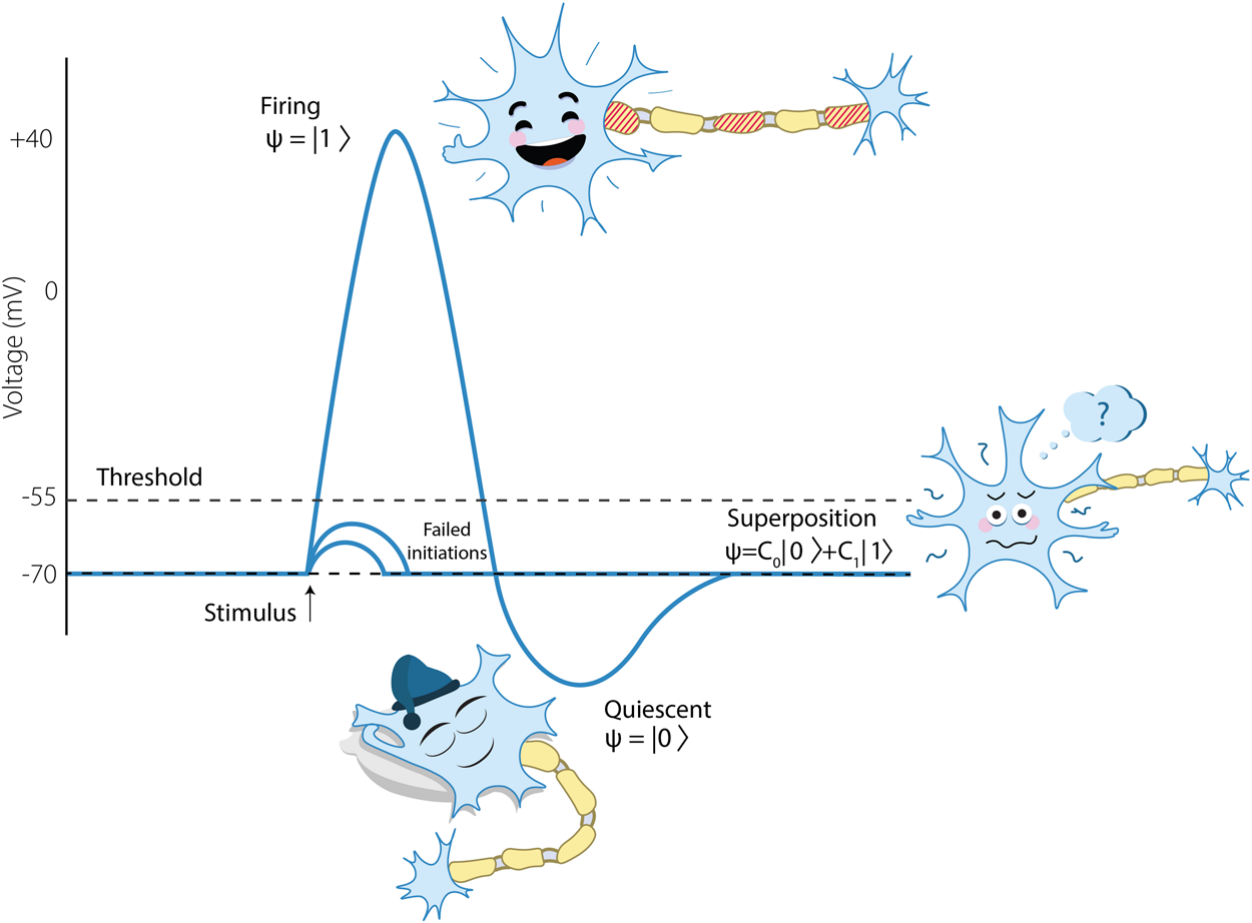
Quantum-like representation of neuron’s informational states.

Here we make a short remark about terminology. The standard neural code is based on non-firing/firing states. We make the non-firing state more detailed. The quiescent state is still encoded by 0 and understood as the state of inactivity, i.e., the refractory state. However, besides of firing and quiescent states, a neuron can be in a state of uncertainty characterized by the range of values of the membrane potential, $V\in [\,-\,70,-\,55]$ (see Fig. 1 for illustration). In this state a neuron can generate an action potential with some probability. Uncertainty in generation of action potentials is a consequence of stochastic operation of ion channels. We also point to random failure of generation, conduction, and acceptance of action potentials (by other neurons)^35,36^.

Another distinguishing feature of our model is that a neuronal group, say *G*, performing some psychological function *F* is an open quantum information system. It interacts with *the surrounding electrochemical environment E*, which also can be modeled with the operational quantum information approach. Interaction of a neuronal group *G* performing the *F*-task with *E* is modeled at the information level by using the quantum master equation.

The process of decision making is described as decoherence of the quantum(-like) information state of *G* in the basis of eigenstates of *F*. A decision state $\bar{\rho }$ is a *steady state* of the dynamics *ρ*(*t*) of the *G*-state; $\bar{\rho }$ is diagonal in the *F*-basis. The existence of steady states is a consequence of interaction with *E*. In short, by our model a group of neurons *G* is ‘working’ on a function *F*, but then convergence for *G* is driven by the broader electrochemical environment treated as an information reservoir.

The steady state $\bar{\rho }$ represents the weights of possible outputs of *F*. In our model a concrete *F*-output is selected by a *classical random generator* with probabilities encoded in the decision state. This is the simplest way to reproduce statistics given by the Born rule. However, there is a plenty of other possibilities to model completion of the process of decision making. We recall that a decision state can be treated as a classical state (for *F*) and a variety of rules of the CP-decision theory can be explored.

In our model even a single neuron can be in a state of superposition representing uncertainty generated by the membrane potential *V*. The main source of this uncertainty is interactions with surrounding neural network (cf.^30^), which may be of huge complexity. Roughly speaking, an isolated neuron is a classical system, cf.^28,29,38^ (but, of course, an isolated bio-system is dead, cf. Schrödinger^37^). Thus the basis of the quantum-like representation for a single neuron is its openness as an electrochemical system.

The crucial feature of the quantum-like representation of information is its linearity. The complex nonlinear dynamics of electrical and chemical flows is lifted to the linear dynamics described, e.g., by *Gorini-Kossakowski-Sudarshan-Lindblad* equation^39^. Here convergence to a steady state is exponentially rapid: state fluctuations are damped by the factors of the form *e*^−*λt*^, where *λ* = *a* + *ib*, *a* > 0. This makes it possible to approach the decision state very quickly. In some sense, the brain transforms the disadvantage of complex fluctuations in information processing (in the form of electrochemical uncertainty) into advantage given by the linear representation. We emphasize that the latter is probabilistic.

We point to another important implication of the use of the quantum-like representation. By coupling some psychological functions to different orthonormal bases the brain is able to realize these functions in the state space of the same group of neurons. Generally, two functions are incompatible (complementary): it is impossible to approach the decision state that is diagonal with respect to both bases. The statistical patterns of such incompatibility in neuronal performance can be found at the behavioral level^6,14–18,20–22,40^.

### Quantum-like superposition from generation of action potentials

We propose to describe uncertainty in generation of an action potential by a neuron, say *N*, by using states’ superposition. Consider two dimensional complex Hilbert space ${\mathcal H}$ (qubit space). At a concrete instant of time neuron’s state can be mathematically described by a superposition of two states, *quiescent and firing*, labeled by |0〉,|1〉:

1 $$\psi ={c}_{0}|0\rangle +{c}_{1}|1\rangle ,\,|{c}_{0}{|}^{2}+|{c}_{1}{|}^{2}=1.$$

It is assumed that these states are orthogonal and normalized, i.e., 〈0|1〉 = 0 and 〈*α*|*α*〉 = 1, *α* = 0, 1. Here the coordinates *c*_0_ and *c*_1_ with respect to the quiescent-firing basis are complex amplitudes representing potentialities for a neuron to be quiescent or firing. Probabilistically these potentialities are expressed by *the Born rule of quantum theory*:

2 $${p}_{\alpha }=|{c}_{\alpha }{|}^{2}=|\langle \psi |\alpha \rangle {|}^{2}.$$

These probabilities can be interpreted statistically. Let *ψ* be a steady state (with respect to the dynamics performing a psychological function). Consider a single neuron *N* and a sufficiently long time interval *T* = *M*Δ, $M\gg 1$, and find the frequency *ν*_1_ = *n*_1_/*M*, where *n*_1_ is the number of Δ-intervals such that *N* produces a spike. Then *p*_1_ ≈ *ν*_1_. We repeat that steady states play the exceptional role in our model as decision states.

This is a good place to mention that the frequentist interpretation of QM was used by von Neumann in book^41^ where he referred to von Mises’ frequency approach to probability, probability as the limit of frequencies in a long series of trials, in a collective^42^. The frequencist interpretation of QM was actively used by one of coauthors^43^, in particular, for demystification of quantum probability. Recently this interpretation was explored in article^44^ in connection with the known law of combining density matrices for subensembles.

This is the ‘observational definition’ of probability. An observer can count spikes. But who is the observer? In our model the brain (more concretely, each psychological function) is a system that is able to perform *self-observations*, detection of information states of neurons and neuronal groups (but not electrochemical states).

### General quantum-like representation of neurons’ states

Neuron’s state space is a complex Hilbert space ${\mathcal H}$ of dimension *m*. In this space we consider the fixed orthonormal basis $|\alpha \rangle ,\alpha =0,\ldots ,m-1$, consisting of states which can be identified by self-observations of some psychological function *F*. Thus each state of a neuron can be represented by superposition

3 $$\psi =\sum _{\alpha }\,{c}_{\alpha }|\alpha \rangle ,\,\sum _{\alpha }\,|{c}_{\alpha }{|}^{2}=1,$$

where complex probability amplitudes *c*_*α*_ represent potentiality of observation of this neuron in the state |*α*〉.

Now consider a group *G* of neurons. The state space of this group is the tensor product ${ {\mathcal H} }_{G}$ of single neuron’s state spaces. Since neurons in *G* interact with each other and with the surrounding electrochemical environment, generally the *G*-state is *entangled*. (The meaning of entanglement here is simply a statement that we cannot decompose the state for two neurons into a product state from each neuron individually).

Consider two neurons and firing/quiescent coding. Generally the state of the compound system *G* has the form

4 $$\psi ={c}_{00}|00\rangle +{c}_{01}|01\rangle +{c}_{10}|10\rangle +{c}_{11}|11\rangle ,$$

where the complex probability amplitudes ${c}_{00},\ldots ,{c}_{11}$ represent the potentiality of the compound system *G* to occupy one of the states $|00\rangle ,\ldots ,|11\rangle$. This potentiality is interpreted probabilistically, e.g., *p*_11_ = |*c*_11_|^2^ is the probability of both neurons firing. Superposition (4) represents correlated behavior of two neurons in *G*. Consider, for example, the state of the form:

5 $$\psi =\frac{1}{\sqrt{2}}(|00\rangle +|11\rangle )$$

(this is one of the Bell states, see (6). A couple of neurons in these state fire and relax simultaneously (up to the time window Δ).

### Example of incompatible representations: distinguishable versus indistinguishable neurons

In the same Hilbert state space, there can be selected a variety of orthonormal bases which can be used by various psychological functions. Hence, the same neuronal group can perform a variety of generally incompatible psychological functions. We illustrate this possibility with a simple example.

Consider two neurons, $G=\{{{\mathscr{N}}}_{1},{{\mathscr{N}}}_{2}\}$ in the state (5). In this state the neurons in *G* are indistinguishable. We stress that this is *information indistinguishability*. Physically, ${{\mathscr{N}}}_{1}$ and ${{\mathscr{N}}}_{2}$ are distingushable: they are located in different places, they have different geometry of connections and so on. Thereby such quantum states match perfectly *the ensemble neural coding with indistinguishable neurons*. To model quantum-like information processing by indistinguishable neurons, we represent the state space ${ {\mathcal H} }_{G}$ as the direct sum of the symmetric and anti-symmetric tensor product spaces: ${ {\mathcal H} }_{G}={ {\mathcal H} }_{G}^{s}\oplus { {\mathcal H} }_{G}^{as}$. Permutation in any pair of neurons does not change vectors belonging to ${ {\mathcal H} }_{G}^{s},$ but it changes the sign for vectors beloning to ${ {\mathcal H} }_{G}^{as}\mathrm{.}$ We illustrate this decomposition in the four-dimensional case ${ {\mathcal H} }_{G}= {\mathcal H} \otimes  {\mathcal H}$. Consider the orthonormal basis known in quantum information theory as the Bell basis:

6 $${\psi }_{1}=\frac{1}{\sqrt{2}}(|00\rangle +|11\rangle ),\,{\psi }_{2}=\frac{1}{\sqrt{2}}(|00\rangle -|11\rangle ),\,{\psi }_{3}=\frac{1}{\sqrt{2}}(|01\rangle +|10\rangle ),$$

7 $${\psi }_{4}=\frac{1}{\sqrt{2}}(|01\rangle -|10\rangle ).$$

It is clear that the states *ψ*_1_, *ψ*_2_, *ψ*_3_ are invariant with respect to permutations of the neurons. This is the basis in ${ {\mathcal H} }_{G}^{s}\mathrm{.}$ Thus any state of the ensemble of two (informationally) indistinguishable neurons can be represented as superposition of these states. We remark that *ψ*_4_ is the only anti-symmetric state, so here ${ {\mathcal H} }_{G}^{as}$ is one-dimensional.

It is convenient to work in *the Fock representation*. For 0/1 coding (quiescent/firing), this is a representation based on counting the number of firing neurons. The basis of the Fock space consists of the states $|n\rangle$, *n* = 0, 1, 2. (big brackets are used to distinguish Fock states from neurons’ states |*αβ*〉, *α*, *β* = 0, 1). Here

8 $$|0\rangle =|00\rangle =\frac{1}{\sqrt{2}}({\psi }_{1}+{\psi }_{2}),\,|1\rangle ={\psi }_{3},\,|2\rangle =|11\rangle =\frac{1}{\sqrt{2}}({\psi }_{1}-{\psi }_{2}).$$

So, working in the Fock representation we are concerned only with the number of firing neurons. Each state $\psi \in { {\mathcal H} }_{G}^{s}$ can be represented in the form of superposition:

9 $$\psi ={d}_{0}|0\rangle +{d}_{1}|1\rangle +{d}_{2}|2\rangle ,$$

where ${\sum }_{n}\,|{d}_{n}{|}^{2}=1.$ Thus if the configuration of two indistinguishable neurons is prepared in the state *ψ* sufficiently many times, then relative frequency of finding *n* = 0, 1, 2, firing neurons approximately equals to *P*_*n*_ = |*d*_*n*_|^2^, (This is again the (self-)observation interpretation of probability.)

### Quantum-like representation of psychological functions

Consider the space of quantum-like states of the group of neurones *G* involved in realization of some psychological function *F*. We proceed with modelling both possibilities: distinguishable and indistinguishable neurons.

Mathematically a psychological *F* is coupled to some fixed orthonormal basis {|*α*〉} in the space of quantum-like states. This basis may be considered as a quantum information representation of the concrete neural code. Suppose that *F* has values ${f}_{1},\ldots ,{f}_{s}$ (e.g., just two values ± 1). It is assumed that each value *f*_*j*_ is coupled to the corresponding set of basis vectors (neuronal states) ${A}_{f},f={f}_{1},\ldots ,{f}_{s}$. Thus *F* is mathematically represented by decomposition of the basis in disjoint blocks *A*_*f*_. Formally *F* can be written as the Hermitian operator of the form:

10 $$F=\sum _{f}\,f\,\sum _{\alpha \in {A}_{f}}\,|\alpha \rangle \langle \alpha |.$$

We remark that the expression

11 $${\pi }_{f}=\sum _{\alpha \in {A}_{f}}\,|\alpha \rangle \langle \alpha |$$

has the mathematical meaning of orthogonal projector on the subspace *L*_*f*_ generated by basis vectors from the block *A*_*f*_. Therefore formally the psychological function can be written in the form: $F={\sum }_{f}\,{f}_{j}{\pi }_{f}.$ However, in our model the process of information processing by neurons is based on the concrete states of the neuronal group *G* performing *F*. Thus the concrete decomposition of *π*_*f*_, see (11), plays an important role in modeling of the functioning of *F*. We remark that in quantum theory selection of the concrete basis can be interpreted as selection of the context of measurement. Thus a psychological function is represented not simply by a Hermitian operator, but by an orthonormal basis (or more precisely, by its decomposition into disjoint blocks corresponding to the values of *F*).

In the simplest case *F* is represented by the Hermitian operator with non-degenerate spectrum, i.e., there is one-to-one correspondence between the *F*-values and basis vectors, $f\to |{\alpha }_{f}\rangle .$ In this case, $F={\sum }_{f}\,f|{\alpha }_{f}\rangle \langle {\alpha }_{f}|.$ However, generally the *F*-operator is degenerate. Take say 100 neurons and 0/1 neural code. Then the corresponding space of quantum-like states has the dimension *D* = 2^100^.

## Discussion

We presented a quantum-like model of processing of information by a group of neurons interacting with the surrounding electrochemical environment. The crucial element of the model is encoding uncertainty in generation of action potentials by superpositions of discrete states representing clusters of electrochemical states (classical or genuine quantum). Such superposition representation of uncertainty can be lifted to generation of QP-features of outputs of a psychological function *F* that are reflected in statistical data collected in cognitive psychology, game theory, social and political sciences, e.g.^6,13–21^. A psychological function is coupled to the fixed basis in the neuronal state space and in principle the latter can be coupled to some classical neural code. From this viewpoint, the complex problem of the neural code is resolved by recognizing that the brain can work with a variety of ‘quantum-like neural codes’ corresponding to orthonormal bases in the state spaces of groups of neurons working on psychological functions.

The basic feature of the presented model is that even the state of a single neuron can be described as a quantum-like state. In the real situation of neuronal network, such network interacts with the neuron by neurotransmitters, which modify the membrane potential of the neuron’s cell body. In other words, the neuron’s potential is influenced by the inputs from other neurons. The modified potential changes the probability of firing of this neuron. This effect can be called ‘uncertainty’. Then we can treat one neuron as a quantum-like machine. The further analysis of information processing of a neuron or neuronal network becomes simple (and, in particular, linear).

In future we plan to generalize the present quantum-like model of brain’s functioning by taking into account the structure of the electrochemical environment, especially the role of neurotransmitters in creation of the superposition representation of information states processed by neurons. At the same time our model can be treated more abstractly, namely, as a quantum-like model of artificial intelligence, cf. Briegel^45^.

## Methods

### Density operators as signatures of environments

The theory of open quantum systems requires us to consider not only pure states of *G*, but also states given by density operators. (A density operator *ρ* is a Hermitian positively semidefinite operator with unit trace.) Each pure state (given by a normalized vector *ψ* of the state space, i.e., 〈*ψ*|*ψ*〉 = 1) can be represented by the density operator, the orthogonal projector on *ψ*. Typically the *ρ*-state is interpreted as a mixed state, i.e., a state representing a statistical mixture of pure states. This interpretation is quite ambiguous, because the same density operator can be represented as a mixture of different ensembles of states. We use the interpretation related to the *Naimark’s dilation theorem* by which each density operator *ρ* (describing the state of some system *S*) can be obtained as a partial trace of a pure state of a larger system, composed of *S* and its environment. The trace is taken with respect to the degrees of freedom of the environment. Then, following D’Ariano^3^ we interpret a pure state as an informationally complete state. Generally, a state given by a density operator is informationally incomplete, since it contains the impact of some environment and a variety of environments may generate the same *ρ*-state.

### Information dynamics of open neuronal systems

In quantum information theory the dynamics of the state of an open system is typically described by the *Gorini-Kossakowski-Sudarshan-Lindblad* (GKSL) equation^39^. It can be written in the form

12 $$\frac{d\rho }{dt}(t)=-\,[H,\rho (t)]+\gamma L\rho (t),\,\rho (0)={\rho }_{0},$$

where *H* is a Hermitian operator (Hamiltonian) acting in the state space of a system (in our case a group of neurons *G* ‘working’ for a psychological function *F*) and *L* is a linear operator acting in the space of linear operators (such maps are often called *super-operators*). In quantum physics, typically the operator *H* represents the state dynamics in the absence of outer environment. (It is assumed that *H* is positively defined.) Generally, Hamiltonian *H* can also contain effects of the environment. The superoperator *L* has to map density operators into density operators, i.e., it has to preserve ‘Hermitianity’, positive definiteness, and the trace. For the present paper, its concrete form is not important, see, e.g., work^39^. The real parameter *γ* > 0 is the coupling constant; it represents strength of (information) interaction with environment ${\mathcal E}$. Generally both *H* and *L* depend on the initial state of the environment and its interaction with neurons (described in the information framework).

In quantum physics the dynamics of an isolated system is described by the Schrödinger equation. For a biological system, it seems to be meaningless to use the notion of an isolated system, even in the approximate setting. Therefore the operator *H* in equation (12) has to be treated on the equal grounds with the superoperator *L*, i.e., as representing (information) interaction with the environment.

The GKSL-equation is a linear ordinary differential equation with constant coefficients. For such an equation, one can quite easily understand whether its solutions ‘stabilize’ to a steady state (by writing it in the vector form and finding the corresponding eigenvalues). Stabilization of *ρ*(*t*) is understood as damping of fluctuations around the final (stable) state. In the mathematical model this is the limiting process $\bar{\rho }={\mathrm{lim}}_{t\to \infty }\,\rho (t).$ However, in reality fluctuations may become negligibly small quite fast. Suppose that function *F* operates with fluctuations’ threshold *ε* > 0. Then the exponentially decreasing factors in a solution very quickly approach *ε*.

### Probabilistic functioning of psychological functions

Consider some psychological function *F* and its representation (10) with the basis {|*α*〉}. Let $\bar{\rho }$ be a steady state for the neuronal information dynamics (modeled mathematically with, e.g., equation (12)) and let it be *diagonal* in the *α*-basis. Thus $\bar{\rho }$ can be considered a classical state (for the psychological function *F*), cf. Zurek^46^. Now we can present the frequentist interpretation of the probabilities encoded in diagonal representation of $\bar{\rho }$ (its Schatten decomposition),

13 $$\bar{\rho }=\sum _{\alpha }\,{p}_{\alpha }|\alpha \rangle \langle \alpha |,$$

where *p*_*α*_  ≥  0, and $${\sum }_{\alpha }\,{p}_{\alpha }=1.$$

*The state* $\bar{\rho }$ *represents statistics of realization of the basis states* {|*α*〉} *in the time series of self-observations on the neuronal group G involved in realization of the psychological function F*.

Thereby, we reserve the statistical mixture interpretation only for the Schatten decomposition, see^47^ for the motivation based on entropic considerations.

The psychological function *F* is modeled as an observer for the neuronal group *G*. Since, for this psychological function, the steady state $\bar{\rho }$ is classical, observations performed by *F* can be modeled as classical monitoring of the state of *G*.

Consider again some time window Δ. This is the ‘interval of state determination’: by monitoring *G* during the time window Δ the psychological function *F* reports (to itself) that the *G*-state is |*α*〉. Consider now some period of time $T=M{\rm{\Delta }},\,M\gg 1.$ The *F* monitors the *G*-states during this period. Suppose that the *α*-state was observed *n*_*α*_ times. Define the relative frequency: ${\nu }_{\alpha }=\frac{{n}_{\alpha }}{{\sum }_{\beta }\,{n}_{\beta }}.$ Then by the frequentist interpretation *p*_*α*_ ≈ *ν*_*α*_. We remark that this probabilistic representation is used in statistical signal processing in neuroscience.

Thus *F* approaches the steady state with the Schatten decomposition corresponding to the basis *F*-eigenstates. Then *F* determines the frequency probability distribution *p* = {*p*_*α*_} encoded in this steady state. This probability distribution is easily transformed into the probability distribution of the possible outcomes of *F*: ${p}_{F}(f)={\sum }_{\alpha \in {A}_{f}}\,{p}_{\alpha },$ see decomposition (10) of *F*. Formally

14 $${p}_{F}(f)={\rm{Tr}}\bar{\rho }\,{\pi }_{f},$$

where *π*_*f*_ is the projector corresponding to the value *f*. But the latter is only a formal mathematical representation. The *F*-neural code is represented by the basis {|*α*〉} and *F* monitors appearance of these basis states.

Finally, *F* should select one concrete outcome *f*. Since the probability distribution *p*_*F*_ is classical, we are now in the framework of classical theory of decision making. In principle any decision rule of this theory can be used. The quantum statistics, given by Born rule (14), is preserved if finalization of *F*-functioning is based on the use of classical random generator with the probability distribution *p*_*F*_. Such random generator can be easily realized through some electrochemical process in the brain.

One can relate this final step with the *free will*. Even if, for some value *f*, probability ${p}_{F}(f)\ll 1,$ there is still a chance that *F* may select this alternative. (On the other hand, if *F* were working, e.g., with odds, it would never select this output.)

### Quantum information versus physical states of neurons

After presenting our quantum-like model of decision making by brain’s modules called psychological functions, we come back to the deep foundational question of the interrelation between quantum informational and physical states. Most quantum information theorists mentioned by the article take the viewpoint that “quantum states” are not the physical states of the quantum objects, but are part of the mathematical machinery (based on Born’s rule) for predicting the probabilities or statistics (when the experiment is repeated many times) of the outcomes of possible quantum measurements, in which, once the measurement is performed, a given outcome is observed. The physical state of say a photon is not so different from a classical state. (There are additional complexities, but they are not important for the moment.) We can say that quantum states are epiphenomenal to the physical reality of the relevant objects. Physical processes of interaction of micro-systems with measurement devices or (by expressing the viewpoint of theory of open quantum systems) with surrounding environment organize themselves exactly so that the quantum theory provides the adequate description in some nonphysical information space.

In some information interpretations, especially QBism, the role of observer is highlighted. QBism’s emphasis of this role matches well with the QP approach to decision making^48^: humans are considered as observers performing self-measurements by answering questions and solving problems. Humans assign subjective probabilities to possible outcomes of self-measurements. However, by closing the gap between electrochemical processes in the brain and the QP-structure of humans’ decision making, we do not imply the presence of a subjective entity, such as Ghost in the machine^49^. And here it is important to note that not all quantum information interpretations are so strongly based on the subjective component, that is, corresponding to a picture of an agent simulating quantum probabilities. In the quantum information approach probabilities can be interpreted objectively; in particular, by using the decoherence viewpoint on generation of steady states and their interpretation as presenting classical frequency probabilities.

In our quantum-like model a psychological function *F* expresses the subjective side of brain’s functioning - it generates probabilities *p*_*f*_. They can be treated in the same way as those subjective probabilities which are considered in subjective expected utility theory or prospect theory. However, the form of the functioning of *F* is based on the electrochemical processes in the brain. And in the previous sections we described the mechanism of transition from electrochemical states to the quantum-information states. Each such state represents a cluster of electrochemical states of a neuron and surrounding electrochemical environment *E*. To describe this transition mathematically is difficult if possible at all. Electrochemical processes in the brain organize themselves to guarantee stable dynamical processing in some nonphysical quantum information space, so to say the mental space. This dynamics matches the abstract mathematical QP model, which has received plenty of confirmation from experimental data collected in cognitive psychology and decision making, see introduction. We use the decoherence model of generation of steady states representing subjective probabilities. The absence of the explicit mathematical description of the mapping of clusters of electrochemical states into quantum-like superpositions does not diminish the significance of outlining (using function *F*) a physical basis for how quantum-like mental representations and corresponding uncertainty relations can emerge, from the action potentials by neurons (cf. Marr^50^).
